# Genetic Relationship and Diversity of *Ellochelon vaigiensis* in the Vietnamese Mekong Delta Using Cytochrome b Gene Sequences

**DOI:** 10.1002/ece3.71639

**Published:** 2025-06-18

**Authors:** Quang Minh Dinh, Tran Thi Huyen Lam, Gieo Hoang Phan, Ton Huu Duc Nguyen

**Affiliations:** ^1^ Faculty of Biology Education School of Education, Can Tho University Can Tho City Vietnam; ^2^ Center for Management of Laboratories ‐ Practices Cuu Long University Vinh Long Vietnam; ^3^ Faculty of Agriculture and Rural Development Kien Giang University Kien Giang Vietnam

**Keywords:** *Cytb* gene sequence, genetic difference, phylogeny, square‐tailed mullet

## Abstract

Square‐tailed mullet (*Ellochelon vaigiensis*) is a monotypic species of the genus *Ellochelon*; however, due to morphological similarities between mullet species, determining their genetic relationship and diversity provides valuable information for classification. The genetic diversity of the square‐tailed mullet collected at four Vietnamese Mekong Delta (VMD) sites was examined by sequencing *Cytb* gene sequences. Five haplotypes were recorded from 12 *Cytb* gene sequences of 792–843 bp long, including 786 invariant (monomorphic) positions and six polymorphic positions. The square‐tailed mullet had a high haplotype diversity (Hd = 0.79) and low nucleotide diversity (*π* = 0.004). Low intraspecific genetic difference was recorded in *E. vaigiensis* collected in sampling sites (0.3%–0.5%). Phylogeny analysis indicated that all square‐tailed mullets belonged to one provisional lineage and were separated from the control square‐tailed mullet lineage. More studies on the lineage of *E. vaigiensis* in VMD are necessary, thereby providing scientists with information about the genetic diversity of this species in the Mekong Delta.

## Graphical abstract

This study investigated the genetic diversity and phylogenetic relationships of *Ellochelon vaigiensis* in the Vietnamese Mekong Delta using cytochrome b (*Cytb*) gene sequences. Analysis of 12 samples from four locations revealed high haplotype diversity (Hd = 0.79) but low nucleotide diversity (*π* = 0.004), with minimal genetic differentiation among populations. Phylogenetic analysis suggested that *E. vaigiensis* in this region may represent a distinct lineage, highlighting the need for further research on its genetic structure and conservation.

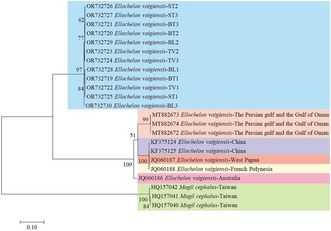

## 1 Introduction

The Mugilidae family is taxonomically diverse and adaptable to habitats in tropical, subtropical, and temperate waters, consisting of approximately 27 genera and 77 identified species (Froese and Pauly 2024). Mullet species of the family Mugilidae are distributed worldwide; they are found in marine, estuarine, and freshwater at all latitudes but have not been found in the Polar Regions (Thomson 1997); a few spend their entire lives in freshwater (Nelson et al. 2016). They can tolerate a wide range of salinities, often entering lagoons and estuaries and then migrating back to the sea during the spawning period. Among them, the square‐tailed mullet (*Ellochelon vaigiensis*) is the monotypic species in the genus *Ellochelon*, along with *Plicomugil* and *Squalomugil*, two other monotypic genera belonging to Squalomugilini. *Ellochelon vaigiensis* can be distinguished from other mullet species based on morphological criteria such as broad head, without adipose eyelids, truncated caudal fin, etc. (Xia, Durand, et al. 2016). Worldwide, this species invades freshwater ecosystems during the rainy season (Nelson et al. 2016; Durand et al. 2017). Juvenile fish can be found in rice fields and mangroves (Harrison and Senou 1997). The square‐tailed mullet is an essential source of food and income for coastal and island communities in the Indo‐Pacific region (Mohanty et al. 2015; Durand et al. 2017). In addition, they are morphologically identified by the following characteristics: pectoral fins are black in juveniles and yellow lower edges in adults; other fins are dark in color; the corners of the mouth have no folds and have labial teeth; with fewer than 28 week and relatively large comb‐like scales on the lateral ranges and distinct dark vertical bands on the lateral scales (Xia, Durand, et al. 2016). Mitochondrial DNA markers can be used to evaluate the genetic diversity and phylogenetic analyses of fish species populations (Xia, Guo, et al. 2016; Parmaksiz 2023). *Cytb* gene which is an effective molecular marker and has been used to analyze genetic data of several species (Maltagliati et al. 2010; Liu et al. 2013; Deng et al. 2014; Parmaksiz 2021).

The phylogeny of grey mullet remains challenging; although various morphological characters have been used, the results are always contradictory and do not provide any convincing answers (Aurelle et al. 2008). Due to substantial morphological conservatism, the differentiation of mullet species poses a challenge. Consequently, field guides often provide an inadequate representation of mullet species (Durand et al. 2017). Therefore, many studies have been conducted to clarify phylogenetic relationships and identify mullet species using molecular methods (Erguden et al. 2010; Liu et al. 2010). Aurelle et al. (2008) investigate the molecular phylogenetic relationships among 5 genera and 12 species of Mugilidae using mitochondrial cytochrome b sequences and 16S rDNA sequences. The results show that all *Mugil* genera form a separate group, while the separation of *Liza*, *Chelon*, and *Oedalechilus* still requires further study. According to recent research, three lineages of *E. vaigiensis* have been identified: one in the Indo‐Pacific (Polynesia and Taiwan); one along the coast of Australia; and one in the Northern Indian Ocean, based on nucleotide polymorphisms of Cytochrome c oxidase 1 and Cytochrome b. Although genetic differences exceed the threshold within species, there are no morphological‐anatomical differences among them. These molecular outcomes suggest that *E. vaigiensis* is a cryptic species complex (Alavi‐Yeganeh et al. 2023). In this study, the phylogenetic relationships of square‐tailed mullets in the Vietnamese Mekong Delta (VMD) are performed based on *Cytb* gene sequence variation analysis. At the same time, genetic differences and parameters of *E. vaigiensis* are analyzed to help assess the current status of this species in the study area.

## 2 Materials and Methods

### 2.1 Sample Collection and Classification

**FIGURE 1:**
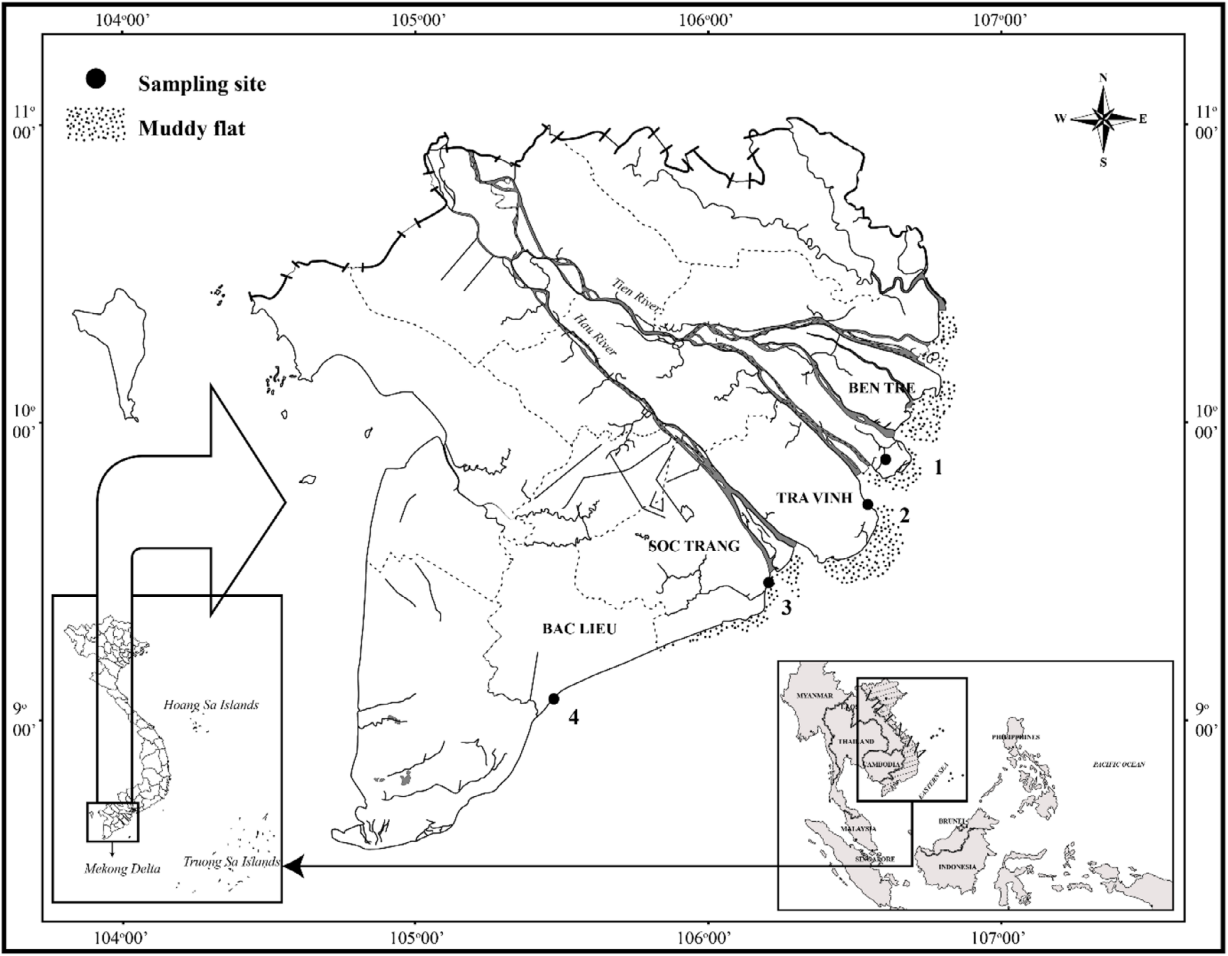
The *Ellochelon vaigiensis* sampling sites in the Vietnamese Mekong Delta (•: Sampling site; BL: Dong Hai‐Bac Lieu; ST: Tran De‐Soc Trang; TV: Duyen Hai‐Tra Vinh; BT: Thanh Phu‐Ben Tre; modified from Dinh (2018)).

A total of 12 squaretail mullet samples were collected from four provinces along VMD (3 identified mullet samples per sampling site) (Figure 1) in October 2021. Twelve fish samples were selected, representing four sampling sites (3 samples/site). Mullet samples were collected by trawling at high tide and retrieved after 2 h. These sites were chosen for sampling because this species has been reported to be present in these areas in previous studies (Dinh, Truong, Nguyen, Lam, et al. 2022; Dinh, Truong, Nguyen, Tran, et al. 2022; Dinh et al. 2024).

Morphologically, all fresh mullet samples were identified using an identification key, according to Tran et al. (2013). After anesthetizing with MS222, the mullet fin clips were dissected, and each fin sample was placed separately in a tube containing cold 90% alcohol. These samples were frozen at −20°C in the laboratory until genetic analysis. These fish samples were processed following the animal welfare assessment code BQ2020‐05/KSP.

### 2.2 Molecular Methods

According to the instructions, genomic DNA was extracted from fin clips using the TopPURE Genomic DNA Extraction Kit (ABT, Vietnam).

A segment of the cytochrome b (*Cytb*) gene was amplified by polymerase chain reaction (PCR) using the primers GluMuq1‐F/Mixcyto937‐2R (Durand et al. 2012) designed by PHUSA Genomics, Vietnam.

GluMug‐1F 5′‐GGCTTGAAAAACCACCGTTG‐3′.

MixCytob937‐2R 5′‐GGKCGGAATGTYAGGCTTCG‐3′.

Each PCR reaction contained 5 μL PCR buffer 10X, 1.5 μL of each primer (10 pmol/μL), dried EZ Mix (PHUSA Genomics), 2 μL genomic DNA, and 40 μL DEPC water until 50 μL. The PCR reaction was carried out according to the following steps: preliminary denaturation: 5 min at 95°C; 35 cycles of 30 s at 95°C, 30 s at the annealing temperature of 55°C, and 45 s at 72°C; final extension: 5 min at 72°C; and kept at 25°C.

Clear and appropriately sized PCR product bands were purified using a PCR Purification Kit (Jena Bioscience, Germany). The procedure was applied according to the manufacturer's recommendations. Sequencing was performed by PHUSA Genomics, Vietnam (https://phusagenomics.com/) using the Sanger method (Sanger et al. 1977) on ABI 3500 (Thermofisher). The *Cytb* gene sequences were sequenced bidirectionally and then assembled into a sequence. All sequences have been registered in GenBank (Table 1). Table 1 also provided information on *Cytb* sequences used as control samples in subsequent analyses.

**TABLE 1:** List of GenBank accession numbers.

Samples	Notes	Accession number
*Ellochelon vaigiensis‐*BT1	Study sample	OR732719
*Ellochelon vaigiensis‐*BT2		OR732720
*Ellochelon vaigiensis‐*BT3		OR732721
*Ellochelon vaigiensis*‐TV1	Study sample	OR732722
*Ellochelon vaigiensis‐*TV2		OR732723
*Ellochelon vaigiensis‐*TV3		OR732724
*Ellochelon vaigiensis‐*ST1	Study sample	OR732725
*Ellochelon vaigiensis‐*ST2		OR732726
*Ellochelon vaigiensis‐*ST3		OR732727
*Ellochelon vaigiensis‐*BL1	Study sample	OR732728
*Ellochelon vaigiensis‐*BL2		OR732729
*Ellochelon vaigiensis‐*BL3		OR732730
*Ellochelon vaigiensis*‐China	Control sample	KF375124 KF375124
*Ellochelon vaigiensis*‐The Persian Gulf and the Gulf of Oman	Control sample	MT882672 MT882673 MT882674
*Ellochelon vaigiensis*‐Australia	Control sample	JQ060186
*Ellochelon vaigiensis*‐West Papua	Control sample	JQ060187
*Ellochelon vaigiensis*‐French Polynesia	Control sample	JQ060188
*Mugil cephalus* ‐Taiwan	Outgroup	HQ157040 HQ157041 HQ157042

*Note:* BT: Thanh Phu‐Ben Tre; TV: Duyen Hai‐Tra Vinh; ST: Tran De‐Soc Trang; BL: Dong Hai‐Bac Lieu.

### 2.3 Data Analysis

The quality of the nucleotide sequences in each sequence was manually checked using the FinchTV 1.4.0 software (http://www.geospiza.com), and then the interfered sequences were edited and cut before proceeding with further analysis. In MEGA 7, the sequences were aligned and analyzed for Kimura 2‐parameter (K2P) genetic distance values among squaretail mullet samples at four sampling sites (Kumar et al. 2016).

Diversity indices such as the number of haplotypes and nucleotide diversity were estimated in DnaSP6 software using the “Gene flow and genetic differentiation” tool (Rozas et al. 2017). The phylogeny of *E. vaigiensis* was described by phylogenetic analysis based on maximum likelihood (ML) implemented in MEGA 7 with 1000 bootstrap replicates. The alternative model Hasegawa‐Kishino‐Yano + G was applied because this model had the smallest Bayesian information criterion (BIC = 7165.087) (Posada and Buckley 2004). Eight *Cytb* gene sequences of *E. vaigiensis* with accession numbers KF375124—KF375125, MT882672—MT882674, JQ060186—JQ060188 were used as control samples for phylogenetic analysis, and all collected in areas other than the Mekong Delta (information of the control samples was presented in Table 1). Three *Cytb* gene sequences of *Mugil cephalus* collected in Taiwan with accession numbers HQ157040—HQ157042, were used as outgroups.

## 3 Results

### 3.1 PCR Amplification

The amplification results of the *Cytb* gene region with the primer pair GluMuq1‐F/Mixcyto937‐2R were approximately 1045 bp in size. In general, the DNA bands on the gel were clear without excess bands, demonstrating that the primer pair explicitly amplified the *Cytb* gene sequence. The negative control sample did not have bands, indicating the DNA was not contaminated. After purification, all of these PCR products were sent for sequencing at PHUSA Genomics.

### 3.2 Sequence Variation

The length of the *Cytb* gene sequences ranged from 792 bp to 843 bp, depending on the samples. The 12 *Cytb* gene sequences obtained had 786 invariable (monomorphic) sites, six variable (polymorphic) sites, and six parsimony‐informative sites.

### 3.3 Genetic Distance and Diversity

Based on the K2P genetic distance values in Table 2, *E. vaigiensis* samples had low and similar values for genetic intra‐and inter‐population variabilities (0.3%–0.5%). Thus, square‐tailed mullet samples collected from BT, TV, ST, and BL had close genetic relationships with each other. The low genetic differentiation among sites (consistently lower than the differentiation within sites) would be clear evidence for connectivity and a lack of population structure.

**TABLE 2:** Percentage of Kimura 2‐parameter genetic distances for a partial sequence of *Cytb* gene.

*Ellochelon vaigiensis* population	BT	TV	ST	BL
BT	*0.5*	—	—	—
TV	0.4	*0.4*	—	—
ST	0.3	0.4	*0.5*	—
BL	0.4	0.3	0.4	*0.5*

*Note:* Italic values: Genetic distance of the population within sampling site; BT: Thanh Phu‐Ben Tre; TV: Duyen Hai‐Tra Vinh; ST: Tran De‐Soc Trang; BL: Dong Hai‐Bac Lieu.

Further analysis of K2P values revealed that the *E. vaigiensis* in the VMD consisted of BT, TV, ST, and BL and was genetically different from the *E. vaigiensis* samples in China and the Persian Gulf and the Gulf of Oman by about 21%–22%. This genetic distance was equivalent to the interspecific genetic distance even though they are morphologically the same species. Thus, they might be two different *E. vaigiensis* lineages.

The number of *E. vaigiensis* haplotypes was five, of which BL included three haplotypes, and the remaining three sampling sites have two haplotypes (Table 3). Hap1 and Hap2 were more commonly recorded at three sampling sites. Hap3, Hap4, and Hap5 only occurred at one sampling site. The *E. vaigiensis* population had haplotype diversity Hd = 0.79, mean nucleotide differences *K* = 3.00, and nucleotide diversity *π* = 0.004. Meanwhile, these values of the *E. vaigiensis* population in China were *S* (number of segregating sites) = 0, *h* (number of haplotypes) =1, Hd = 0.00, *K* = 0.00, *π* = 0.00; and in the Persian Gulf and the Gulf of Oman were *S* = 1, *h* = 2, Hd = 0.67, *K* = 0.67, *π* = 0.001.

**TABLE 3:** Distribution of haplotypes in the *Ellochelon vaigiensis* population at four sampling sites.

Haplotypes	*Ellochelon vaigiensis* populations			
	BT	TV	ST	BL
Hap1	1	1	1	—
Hap2	2	—	2	1
Hap3	—	2	—	—
Hap4	—	—	—	1
Hap5	—	—	—	1
Total	3	3	3	3

*Note:* BT: Thanh Phu‐Ben Tre; TV: Duyen Hai‐Tra Vinh; ST: Tran De‐Soc Trang; BL: Dong Hai‐Bac Lieu.

### 3.4 Phylogenetic Analyses

**FIGURE 2:**
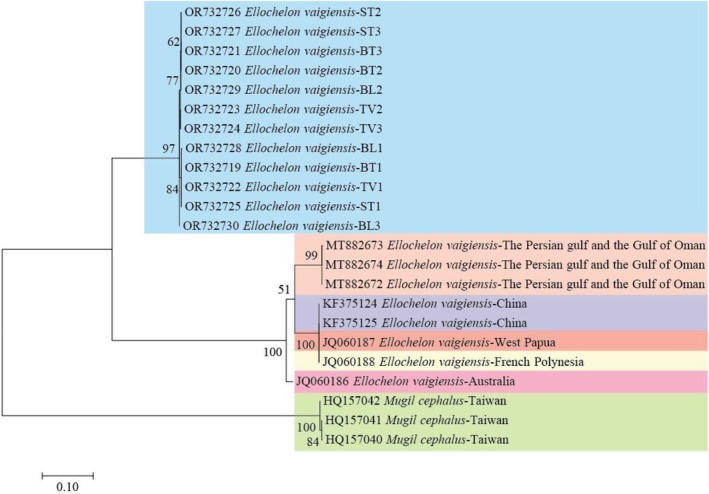
Maximum likelihood tree based on *Cytb* gene sequences employing the Hasegawa‐Kishino‐Yano + G model with the bootstrap test (1000 replicates). The tree is presented on the scale, where branch lengths are measured in the number of substitutions per site (BT: Thanh Phu‐Ben Tre; TV: Duyen Hai‐Tra Vinh; ST: Tran De‐Soc Trang; BL: Dong Hai‐Bac Lieu).

The phylogenetic tree highlighted two supported lineages *E. vaigiensis*, with bootstrap values of high confidence (99%–100%). The outgroup consisting of *M. cephalus* ‐Taiwan samples was separated with a bootstrap value of 100%, supporting the reliability of the phylogeny. All squared‐tail mullet samples collected in VMD belonged to one lineage; meanwhile, the control *E. vaigiensis* samples belonged to a different lineage, possibly due to geographical separation (Figure 2). This result was strongly supported by the fact that the K2P values of fish samples in the study were low and did not differ too much (0.3%–0.5%). These four sampling locations had unclear geographical differences, so all mullet samples in this study may be from the same lineage. Analysis indicated that the current study's *Cytb* gene sequences of the mullet samples were genetically invariable and had low polymorphism.

## 4 Discussion

Sequence analysis of part of the *Cytb* gene sequences of *E. vaigiensis* in VMD showed negligible genetic differences between sampling sites. Most populations had at least two haplotypes, suggesting deficient connectivity between populations, at least concerning female contributions. The reason was that this survey was carried out with mtDNA, which was known to be inherited from the mother. Although mitochondrial DNA had a high evolutionary rate (Caldara et al. 1996), the genetic distance between individuals of the same species was usually as low as 1% (~1%); for example, 1.2% of goby species collected in India (Viswambharan et al. 2015) and 0.39% of fish species in Australia (Ward et al. 2005). The intraspecific genetic distance at the different sampling sites in this study ranged from 0.4% to 0.5%; such genetic distance values among *Cytb* gene sequences were common in marine teleost species (Cárdenas et al. 2005). Similarly, the *Cytb* gene sequence research results of grey mullet samples worldwide showed profound differences between lineages with different geographical origins. The genetic distances (*p*) between *Mugil cephalus* haplotypes ranged from 0.3% to 8.7%, with an average *p* distance of 5.8% (Livi et al. 2011), higher than in this study. This could potentially elucidate the genetic distance values observed in the population of square‐tailed mullets. Employing independent and more slowly evolving nuclear gene markers might prove beneficial in further clarifying the assumption that the *E. vaigiensis* sample collected in VMD was of the same lineage.

The square‐tailed mullet population within the estuaries of VMD exhibited low nucleotide diversity (*μ*) but high haplotype diversity (*h*), indicating a rapid population increase, possibly attributed to the bottleneck effect as discussed by Grant and Bowen (1998). The reason was thought to be expansion after a period of low population size; a rapidly growing population increased the likelihood of maintaining new mutations (Rogers and Harpending 1992). A similar result was found in the *E. vaigiensis* population in the Persian Gulf and the Gulf of Oman; in contrast, the population in China was almost undifferentiated with only 1 haplotype. This showed that the *Cytb* genes of *E. vaigiensis* at these sites were less variable than in VMD. Haplotypes were relatively abundant in VMD, probably due to the increased mutation rate in mitochondrial DNA compared with nuclear protein‐coding loci (Grant and Bowen 1998). Liu et al. (2015) highlighted that most pelagic species exhibited elevated haplotype diversity and diminished nucleotide diversity. In a study investigating geographical relationships between *Mugil cephalus* populations using the *Cytb* gene sequence, most populations showed values of genetic diversity ranging from low to medium (*μ*); for example, the highest haplotype diversity (*h*) was observed in eastern Australia and Hawaii, while lower values of h and relatively higher *μ* were identified in Taiwan and the Sea of Japan (Livi et al. 2011). These results suggested that there may have been a temporary bottleneck in a large area of the ancestral population or that two lineages coexisted. However, increasing the number of samples at this site is necessary to verify this assumption.

The mitochondrial phylogeny of *E. vaigiensis* had long terminal branches and much shorter internal branches. According to Durand et al. (2012), this type of genetic tree showed that either mutation had saturated or radiation had quickly occurred during the initial diversification process. Variations in allele frequencies or genotypes among marine fish populations may be due to migration, genetic flow, and natural selection (Grant et al. 1987). Marine fish populations may typically have little or no genetic differences due to their high gene flow. In this study, all *E. vaigiensis* samples belonged to one lineage and were distinct from the *E. vaigiensis* collected in China (KF375124, KF375125), the Persian Gulf and the Gulf of Oman (MT882672, MT882673, MT882674), Australia (JQ060186), West Papua (JQ060187), and in French Polynesia (JQ060188). The haplotypes of the square‐tailed mullet population sampled in VMD belonged to a lineage so distinct from the rest of the samples that suspicions of cryptic species were also raised. These might be other coexisting lineages, migration, or lack of gene flow between these populations due to human fishing and fry collection activities. The temporary lineage could be due to migration, disruption of gene flow, or the species' taxonomic status (Durand and Borsa 2015), but further research was needed to confirm these assumptions. Gene flow also contributed to the formation of distinct lineages because gene flow reduced genetic differentiation among populations, thus preventing or slowing the population's evolution into distinct lineages in different geographic areas (Agrios 2005). Similarly, Hasan et al. (2021) documented multiple lineages of *E. vaigiensis* coexisting in a study on DNA barcoding of mullet (family Mugilidae) in Pakistan. Lineage 1 was designated AAU0553 from Pakistan, Iran, Malaysia, and Indonesia, lineage 2 ACK7668 from Australia, and lineage 3 AAC9398 from Indonesia to French Polynesia. Lineage 1 was genetically different from lineages 2 and 3 by 6.2% and 5.8%, respectively. Similarly, the study of Alavi‐Yeganeh et al. (2023) on the *E. vaigiensis* population in the Persian Gulf and the northern Sea of Oman recorded three independent evolutionary lineages of this species. Two lineages were present in the Mugilidae phylogeny, published by Durand et al. (2012) suggested, a lineage noted by Hasan et al. (2021). Alavi‐Yeganeh et al. (2023) believed that lowering sea levels and drastic reductions in habitat area prevented gene flow and created favorable conditions for genetic population bottlenecks in *E. vaigiensis*, causing low intralineage genetic diversity and vital cloning. Another reason could be that changes in seawater temperature, current direction, and eddy strength are likely to cause severe fluctuations in population size. Such climate‐related dynamics could explain the low levels of nucleotide diversity and shallow coalescence of mtDNA phylogenies (Grant and Bowen 1998).

What did the shallow *Cytb* gene population structure and low genetic diversity of *E*. *vagiensis* mean for the conservation of this species? This was clearly a common phenomenon in marine fishes and had thus only been exacerbated in recent decades by deteriorating coastal and ocean habitats (Sherman 1994) and fishing activities. However, rapid evolutionary declines in genetic diversity due to fishing (Smith et al. 1991) and loss of low‐frequency alleles (often not detected by heterozygosity estimates) might be of particular concern for the genetic health of marine species and the maintenance of their evolutionary potential (Ryman et al. 1994). Even tremendous populations could be vulnerable to regional extinction. Most healthy marine fish populations were reduced by overfishing, and in many cases, overfished populations included juveniles. In these cases, a failure to recruit for 3 or 4 years could lead to commercial extinction and the complete extinction of a population in a region. Therefore, management strategies for fisheries, one of the most productive fisheries in VMD, must include activities that protect fragile populations in changing habitats.

## 5 Conclusion

Within the scope of this study, the square‐tailed mullet population in VMD exhibited high haplotype and low nucleotide diversity values based on analysis of *Cytb* gene sequences. *E. vaigiensis* samples collected from VMD had a more significant genetic divergence than control *E. vaigiensis* samples collected from China, the Persian Gulf, and the Gulf of Oman, Australia, West Papua, and French Polynesia. Further studies on the square‐tailed population in VMD should expand the sampling area, increase the sample size, and use other polymorphic molecular markers.

## Author Contributions

**Quang Minh Dinh:** conceptualization (equal), funding acquisition (equal), investigation (equal), methodology (equal), writing – original draft (equal), writing – review and editing (equal). **Tran Thi Huyen Lam:** investigation (equal), methodology (equal), software (equal), writing – original draft (equal), writing – review and editing (equal). **Gieo Hoang Phan:** writing – original draft (equal), writing – review and editing (equal). **Ton Huu Duc Nguyen:** conceptualization (equal), investigation (equal), methodology (equal), writing – original draft (equal), writing – review and editing (equal).

## Ethics Statement

The fish used in this study was approved by the Council for Science and Education of the School of Education, Can Tho University (Animal Welfare Assessment No. BQ2020‐05/KSP).

## Conflicts of Interest

The authors declare no conflicts of interest.

## Data Availability

All data of the present study are uploaded to GenBank to get accession numbers (OR732719; OR732720; OR732721; OR732722; OR732723; OR732724; OR732725; OR732726; OR732727; OR732728; OR732729; OR732730) and presented in Table 1.
